# Prognostic Implication of Platelet Reactivity According to Procedural Complexity After PCI: Subanalysis of PTRG-DES Consortium

**DOI:** 10.1016/j.jacasi.2023.10.011

**Published:** 2023-12-26

**Authors:** Xuan Jin, Young-Hoon Jeong, Kwang Min Lee, Sung Cheol Yun, Byeong-Keuk Kim, Hyung Joon Joo, Kiyuk Chang, Yong Whi Park, Young Bin Song, Sung Gyun Ahn, Jung-Won Suh, Sang Yeub Lee, Jung Rae Cho, Ae-Young Her, Hyo-Soo Kim, Do-Sun Lim, Eun-Seok Shin, Moo Hyun Kim, PTRG-DES Consortium Investigators

**Affiliations:** a Department of Cardiology, Dong-A University Hospital, Busan, South Korea; b Department of Cardiology, Yanbian University Hospital, Yanji, Jilin, China; c CAU Thrombosis and Biomarker Center, Chung-Ang University Gwangmyeong Hospital, Gwangmyeong, South Korea and Department of Internal Medicine, Chung-Ang University College of Medicine, Seoul, South Korea; d Department of Clinical Epidemiology and Biostatistics, University of Ulsan College of Medicine, Asan Medical Center, Seoul, South Korea; e Severance Cardiovascular Hospital, Yonsei University College of Medicine, Seoul, South Korea; f Department of Cardiology, Cardiovascular Center, Korea University Anam Hospital, Korea University College of Medicine, Seoul, South Korea; g Division of Cardiology, Department of Internal Medicine, College of Medicine, Catholic University of Korea, Seoul, South Korea; h Department of Internal Medicine, Gyeongsang National University School of Medicine and Cardiovascular Center, Gyeongsang National University Changwon Hospital, Changwon, South Korea; i Division of Cardiology, Department of Medicine, Samsung Medical Center, Sungkyunkwan University School of Medicine, Seoul, South Korea; j Department of Cardiology, Yonsei University Wonju Severance Christian Hospital, Wonju, South Korea; k Department of Internal Medicine, Seoul National University College of Medicine and Department of Cardiology, Seoul National University Bundang Hospital, Seongnam, South Korea; l Cardiology Division, Department of Internal Medicine, Kangnam Sacred Heart Hospital, Hallym University College of Medicine, Seoul, South Korea; m Division of Cardiology, Department of Internal Medicine, Kangwon National University School of Medicine, Chuncheon, South Korea; n Department of Internal Medicine and Cardiovascular Center, Seoul National University Hospital, Seoul, South Korea; o Division of Cardiology, Ulsan University Hospital, University of Ulsan College of Medicine, Ulsan, South Korea

**Keywords:** clinical outcomes, complex PCI, platelet reactivity

## Abstract

**Background:**

Complex percutaneous coronary intervention (C-PCI) and high platelet reactivity (HPR) have been proposed as representative risk factors for the high ischemic phenotype. Uncertainty remains regarding the relative prognostic importance of these factors.

**Objectives:**

This study aimed to investigate the prognostic implication of HPR according to procedural complexity.

**Methods:**

Patients treated with drug-eluting stent implantation (PTRG-PFT cohort; N = 11,714) were classified according to procedural complexity. HPR criteria were determined using VerifyNow (≥252 P2Y_12_ reaction units). The major adverse cardiac and cerebrovascular events (MACCE) (the composite of all-cause death, myocardial infarction, definite stent thrombosis, or stroke) and major bleeding were assessed for up to 3 years.

**Results:**

C-PCI was performed in 3,152 patients (26.9%). C-PCI significantly increased the risk of MACCE (HR_adjusted_: 1.21; 95% CI: 1.01-1.44; *P* = 0.035), driven by a higher rate of all-cause death (HR_adjusted_: 1.45; 95% CI: 1.15-1.83; *P* = 0.002), although it did not increase the risk of major bleeding. Irrespective of procedural complexity, the HPR phenotype was significantly associated with MACCE (*P*_interaction_ = 0.731) and all-cause mortality (*P*_interaction_ = 0.978), in which the prognostic implication appeared prominent within 1 year. The HPR phenotype did not show a significant interaction with any type of C-PCI. In addition, the number of complexity features per procedure did not proportionally increase the risk of MACCE.

**Conclusions:**

C-PCI was significantly associated with 3-year risk of MACCE and all-cause death. The HPR phenotype appears to have a similar prognostic implication irrespective of the type and extent of procedural complexity. (Platelet Function and Genotype-Related Long-Term Prognosis in DES-Treated Patients [PTRG-DES]; NCT04734028)

Dual antiplatelet therapy (DAPT) with aspirin and a P2Y_12_ inhibitor is the cornerstone of pharmaceutical treatment in patients undergoing percutaneous coronary intervention (PCI) to reduce the risk of ischemic events.^1^, ^2^, ^3^, ^4^, ^5^ The first broadly used P2Y_12_ inhibitor clopidogrel has considerable interindividual variation and achieves inadequate platelet inhibition in the majority of cases.^6^ High on-clopidogrel platelet reactivity (HPR) determined by validated platelet function tests (PFTs) is a well-established risk factor for atherothrombotic events after PCI.^7^, ^8^, ^9^ Therefore, addressing this issue is critical for high-risk patients, including those presenting with acute coronary syndrome (ACS).

The concept of complex PCI (C-PCI) has recently been proposed as a representative factor for high-risk cohorts.^10^ Increasing the atherosclerotic burden and complicated implementations of stent struts in diseased vessels can lead to potential interactions with blood risk factors such as platelet activation. Although the clinical application of potent P2Y_12_ inhibition and/or a prolonged DAPT strategy^11^ has been suggested to prevent the risk of atherothrombotic complications in patients treated with C-PCI,^12^ there have been few dedicated prospective clinical trials in the current era of drug-eluting stents (DES).

The present analysis was performed to evaluate the impact of platelet reactivity on long-term clinical outcomes according to procedural complexity using data from a largescale real-world DES-treated cohort.

## Methods

### Study design and population

The multicenter PTRG-DES (Platelet Function and Genotype-Related Long-Term Prognosis in DES-Treated Patients) consortium is a multicenter, real-world registry of patients in South Korea who have undergone PCI with DES and received DAPT with aspirin and clopidogrel (NCT04734028).^13^^,^^14^ An organizing committee of the PTRG-DES investigators was established to define the scientific goals. The organizing committee invited the lead investigators of clopidogrel-related prospective clinical registries published in www.pubmed.gov as of January 2018 to participate. Criteria for participation included the availability of on-clopidogrel PFT or genotyping data, and for outcome analysis, the availability of baseline characteristics and clinical prognosis in patients treated with DES implantation.

In total, 9 prospective registries enrolling 32 Korean academic centers’ patients have joined the PTRG-DES consortium, contributing data from 13,160 DES-treated patients between July 2003 and August 2018. We obtained 11,714 PFT results measured by the VerifyNow (Accriva Diagnostics) assay (PTRG-PFT cohort) and 8,163 genotyping results relating to clopidogrel responsiveness (PTRG-Genotype cohort). The institutional review board of each participating center approved the registry and waived the requirement for written informed consent for access to their registries. The study was performed in accordance with Good Clinical Practice Guidelines and the principles of the Declaration of Helsinki.

**Figure 1:**
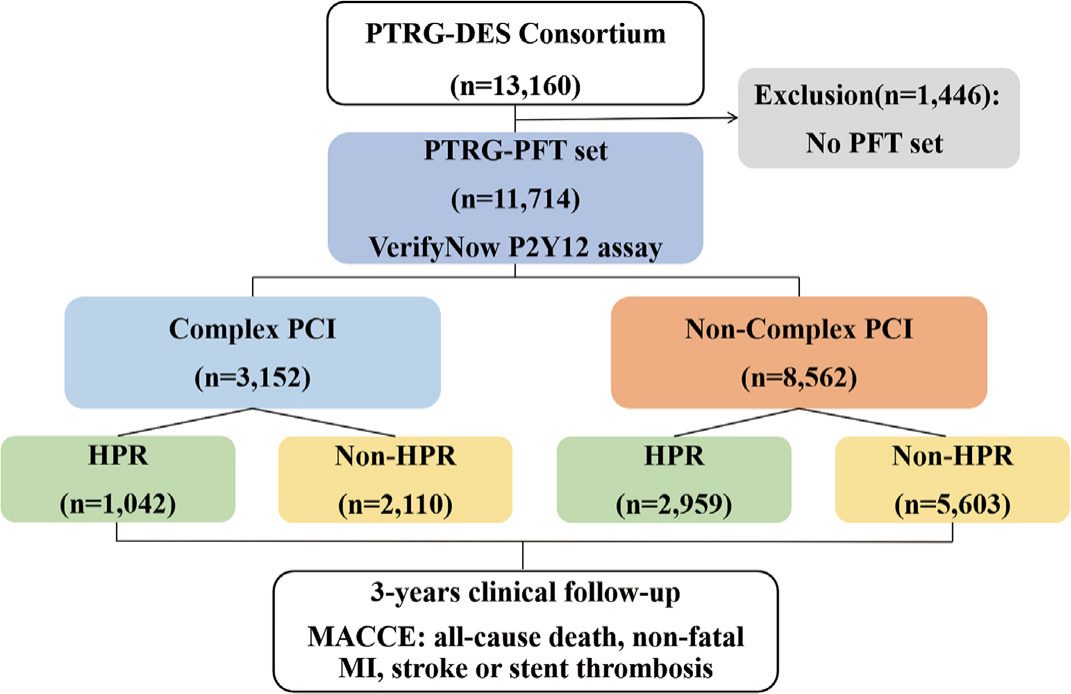
Study Flow Diagram A total of 13,160 participants from the PTRG-DES (Platelet Function and Genotype-Related Long-Term Prognosis in DES-Treated Patients) consortium treated with drug-eluting stents were enrolled between July 2003 and August 2018. Patients in the platelet function test cohort (PFT) (n = 11,714) were classified according to procedural complexity and platelet reactivity. High platelet reactivity (HPR) criteria were determined using VerifyNow (≥252 P2Y_12_ reaction units). The primary endpoint was the occurrence of major adverse cardiac and cerebrovascular events (MACCE) including all-cause death, nonfatal myocardial infarction (MI), definite stent thrombosis (ST), or nonfatal stroke during the 3-year follow-up period. PCI = percutaneous coronary intervention.

Consecutive patients at each center were eligible for enrolment if they had been successfully treated with 1 or more DES approved by the U.S. Food and Drug Administration or with a CE mark, and were adequately loaded (if the patients were not taking aspirin or clopidogrel at the time of PCI, loading doses of aspirin, 300 mg, and clopidogrel, 300 mg to 600 mg, were administered before PCI. After PCI, DAPT with aspirin and clopidogrel for 12 months was recommended, but the discontinuation of DAPT was left to each physician’s discretion) with aspirin and clopidogrel, regardless of lesion complexity. The exclusion criteria were PCI strategies other than DES, and the use of any P2Y_12_ inhibitor other than clopidogrel or oral anticoagulants. The study flow is provided in Figure 1.

### Procedure

All PCI procedures were performed according to standard techniques.^13^ Following procedures, patients were administered with 100 mg of aspirin and 75 mg of clopidogrel daily. Patients were recommended to stay on aspirin treatment indefinitely and clopidogrel for at least 1 year, with all other treatments as per standard of care. Clinical outcomes were evaluated until the last outpatient visit.

### Platelet function test

Platelet reactivity was measured after an adequate period to ensure a full antiplatelet effect, using the VerifyNow P2Y_12_ assay (Accriva Diagnostics).^15^ The protocol followed the manufacturer’s recommendations, with details described previously.^16^ PFT for clopidogrel responsiveness was performed after either: 1) 600-mg loading for at least 6 hours; 2) 300-mg loading for at least 12 hours; or 3) 75-mg maintenance for at least 5 days before PCI. If eptifibatide or tirofiban was used during PCI, a 24-hour washout period was required before VerifyNow testing. No patients receiving abciximab were enrolled due to the long washout period.

On-clopidogrel platelet reactivity was reported in P2Y_12_ reaction units (PRUs). We assessed PRUs as continuous and categorical measures. Additionally, the cutoff of HPR to adenosine diphosphate was defined as ≥252 PRU according to the time-dependent receiver-operating characteristic curve analysis for East Asian patients.^13^

### Definition of complex PCI

In the present analysis, C-PCI was defined according to a modified version of previously published criteria,^3^ which included PCI with at least 1 of the following characteristics: 1) 3 vessels treated; 2) ≥3 lesions treated; 3) ≥3 stents implanted; 4) total stent length >60 mm; 5) bifurcation with 2 stents implanted; 6) left main PCI; and 7) chronic total occlusion PCI.

### Clinical outcomes

The primary endpoint was the occurrence of major adverse cardiac and cerebrovascular events (MACCE) including all-cause death, nonfatal myocardial infarction (MI), definite stent thrombosis (ST), or nonfatal stroke during the 3-year follow-up period. The key secondary endpoints were all-cause death and major bleeding (Bleeding Academic Research Consortium [BARC] type 3-5).^17^

All deaths were considered to be due to cardiovascular causes unless a definite noncardiovascular cause could be established. MI was defined as increased cardiac troponin values with ischemic symptoms, or ischemic changes on electrocardiogram, or imaging evidence of recent loss of viable myocardium, or new regional wall motion abnormalities that were not related to the interventional procedure (type 4a).^13^^,^^18^ ST (definite) was defined according to Academic Research Consortium criteria.^19^ Stroke was defined as evidence of neurological deficit requiring hospitalization and clinically documented lesions on brain computed tomography or magnetic resonance imaging. An independent clinical event committee masked to VerifyNow results adjudicated all clinical events using the original source documents.

### Statistical analysis

The Kolmogorov-Smirnov test was performed to analyze the normal distribution of continuous variables. Continuous variables are expressed as mean ± SD or as median (Q1-Q3), whereas categorical variables are presented as absolute numbers and frequencies (%). Student’s unpaired *t*-test and the Mann-Whitney *U* test were used to evaluate the parametric and the nonparametric continuous variables, respectively. Categorical variables were compared using the Pearson chi-square test or Fisher exact test when the Cochran rule was not met. The cumulative incidence of clinical events up to 3 years was calculated using the Kaplan-Meier method and compared using the log-rank test. HRs with a 95% CI were derived from a Cox regression model. Unadjusted and adjusted Cox proportional hazard models were used to compare the clinical events according to lesion complexity with an adjustment of the important prognostic covariates (age, gender, body mass index, hypertension, dyslipidemia, diabetes mellitus, chronic kidney disease, congestive heart failure). The consistency of HPR between subjects with and without procedural complexity was evaluated through the inclusion of HPR-by-procedural complexity status interaction terms (multiplicative interaction) in a Cox model. The proportional hazards assumption was tested on the basis of Schoenfeld residuals test and proportional hazards assumptions being met.

Statistical significance was set at *P* value <0.05. All statistical analyses were performed using IBM/SPSS v23.0 (IBM/SPSS) and RStudio (Integrated Development Environment for R. RStudio, PBC).

## Results

### Characteristics of patients

From the PTRG-PFT cohort (N = 11,714), most patients were treated with second generation DES (91.9%) (Figure 1). Of those, 26.9% of the total cohort (n = 3,152) were treated with C-PCI and stent length >60 mm was the most frequent type of C-PCI performed (Central Illustration). Compared with the non–C-PCI group, the C-PCI group was older and had a higher prevalence of hypertension, diabetes, and chronic kidney disease. Various features of the procedure were higher in the C-PCI group. Cilostazol was more frequently used for the C-PCI group, whereas statins and proton pump inhibitors were more commonly prescribed in the non–C-PCI group (Table 1). There were no differences in PRU value (218 ± 79 PRU vs 218 ± 78 PRU; *P* = 0.972) and HPR rate (33.1% vs 34.6%; *P* = 0.129) between the groups.

**Central Illustration:**
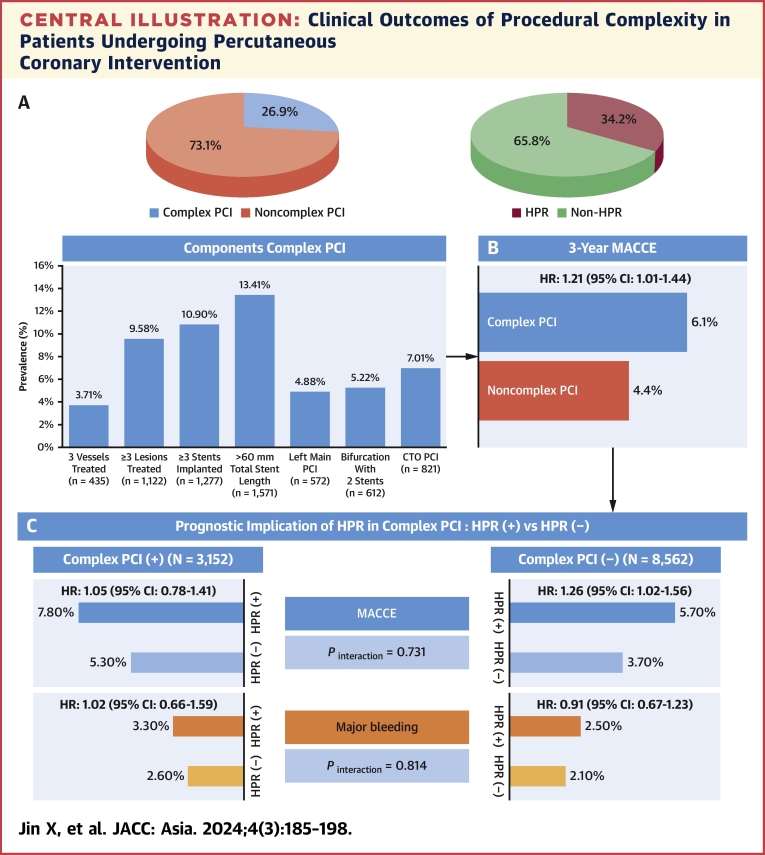
Clinical Outcomes of Procedural Complexity in Patients Undergoing Percutaneous Coronary Intervention (A) Proportion of complex percutaneous coronary intervention (PCI), high platelet reactivity (HPR), and subtype of complex PCI. (B) The cumulative incidence of major adverse cardiac and cerebrovascular events (MACCE) according to complex PCI. (C) Prognostic implication of HPR according to presence of complex PCI.

**Table 1:** Baseline Characteristics of Study Population

	Overall (N = 11,714)	Complex PCI (+) (n = 3,152)	Complex PCI (−) (n = 8,562)	*P* Value
Index presentation				<0.001
Stable angina	4,910 (41.9)	1,366 (43.3)	3,544 (41.4)	
Unstable angina	3,466 (29.6)	940 (29.8)	2,526 (29.5)	
Non–ST-segment elevation MI	1,860 (15.9)	612 (19.4)	1,248 (14.6)	
ST-segment elevation MI	1,478 (12.6)	234 (7.4)	1,244 (14.5)	
Age, y	64.4 ± 10.9	65.0 ± 10.7	64.1 ± 10.9	<0.001
Male	7,951 (67.9)	2,167 (68.8)	5,784 (67.6)	0.219
Body mass index, kg/m^2^	24.5 ± 3.1	24.6 ± 3.1	24.5 ± 3.1	0.393
Risk factors				
Hypertension	7,049 (60.2)	1,989 (63.1)	5,060 (59.1)	<0.001
Dyslipidemia	7,555 (64.5)	2,084 (66.1)	5,471 (63.9)	0.026
Smoking	3,285 (28.0)	884 (28.0)	2,401 (28.0)	0.997
Diabetes mellitus	4,057 (34.6)	1,216 (38.6)	2,841 (33.2)	<0.001
Insulin-treated	367 (3.1)	105 (3.3)	262 (3.1)	0.455
Chronic kidney disease	2,432 (20.8)	721 (22.9)	1,711 (20.0)	0.001
Current dialysis	162 (1.4)	47 (1.5)	115 (1.3)	0.543
Anemia	2,921 (24.9)	864 (27.4)	2,057 (24.0)	0.672
Previous history				
History of peripheral artery disease	1,453 (12.4)	382 (12.1)	1,071 (12.5)	0.571
History of congestive heart failure	880 (7.5)	244 (7.7)	636 (7.4)	0.569
Previous MI	839 (7.2)	256 (8.1)	583 (6.8)	0.015
Previous PCI	1,568 (13.4)	428 (13.6)	1,140 (13.3)	0.710
Previous CABG	150 (1.3)	150 (4.8)	0 (0.0)	<0.001
Previous stroke	813 (6.9)	252 (8.0)	561 (6.6)	0.006
Laboratory measurements				
VerifyNow PRU	218 ± 79	218 ± 78	218 ± 79	0.972
HPR rate	4,001 (34.2)	1,042 (33.1)	2,959 (34.6)	0.129
LV ejection fraction, %	58.8 ± 10.6	57.6 ± 11.0	59.2 ± 10.4	<0.001
WBC, × 10^3^/mm^3^	7.9 ± 3.0	7.8 ± 2.9	7.9 ± 3.0	0.263
Hemoglobin, g/dL	13.6 ± 1.8	13.5 ± 1.9	13.6 ± 1.8	0.005
Platelet, × 10^3^/mm^3^	233.6 ± 72.4	230.0 ± 68.6	235.0 ± 73.7	0.001
GFR, mL/min/1.73 m^2^, MDRD	78.7 ± 27.1	77.9 ± 28.6	79.0 ± 26.4	0.050
HbA_1c_, %	6.6 ± 1.4	6.8 ± 1.5	6.5 ± 1.3	<0.001
Total cholesterol, mg/dL	174.0 ± 44.5	171.9 ± 46.0	174.8 ± 43.9	0.003
LDL-cholesterol, mg/dL	106.8 ± 43.4	106.6 ± 51.7	106.8 ± 39.9	0.752
HDL-cholesterol, mg/dL	44.0 ± 12.8	43.0 ± 11.7	44.3 ± 13.1	<0.001
Triglyceride, mg/dL	143.2 ± 98.3	141.6 ± 93.7	143.8 ± 99.9	0.297
Angiographic feature				
ACC/AHA lesion				<0.001
A/B1 type	5,238 (44.7)	735 (23.3)	4,503 (52.6)	
B2/C type	6,476 (55.3)	2,417 (76.7)	4,059 (47.4)	
Number of diseased vessels				<0.001
One	7,170 (61.2)	1,025 (32.5)	6,145 (71.8)	
Two	3,039 (25.9)	1,315 (41.7)	1,724 (20.1)	
Three	1,505 (12.8)	812 (25.8)	693 (8.1)	
Multivessel disease	4,544 (38.8)	2,127 (67.5)	2,417 (28.2)	<0.001
Bifurcation lesion	1,363 (11.6)	826 (26.2)	537 (6.3)	<0.001
Chronic total occlusion lesion	821 (7.0)	821 (26.0)	0 (0.0)	<0.001
Procedural data				
Multivessel PCI	2,917 (24.9)	1,803 (57.2)	1,114 (13.0)	<0.001
Treated lesions				<0.001
Left main coronary artery	572 (4.9)	572 (18.1)	0 (0.0)	
Left anterior descending coronary artery	6,960 (59.4)	2,068 (65.6)	4,892 (57.1)	
Left circumflex coronary artery	3,434 (29.3)	1,247 (39.6)	2,187 (25.5)	
Right coronary artery	4,460 (38.1)	1,667 (52.9)	2,793 (32.6)	
Stent type				0.547
1st generation DES	944 (8.1)	205 (6.5)	739 (8.6)	
2nd generation DES	10,770 (91.9)	2,947 (93.5)	7,823 (91.4)	
Number of stents, n	1.6 ± 0.8	2.4 ± 0.9	1.3 ± 0.5	<0.001
Stent length, mm	35.9 ± 22.5	58.7 ± 28.3	27.5 ± 11.7	<0.001
Stent diameter, mm	3.02 ± 0.44	2.91 ± 0.44	3.06 ± 0.43	<0.001
Concomitant medications				
DAPT maintenance, day	535 ± 355	576 ± 358	520 ± 353	<0.001
Aspirin	11,409 (97.4)	3,055 (96.9)	8,354 (97.6)	0.051
Clopidogrel	11,714 (100.0)	3,152 (100.0)	8,562 (100.0)	1.000
Cilostazol	1,219 (10.4)	403 (12.8)	816 (9.5)	<0.001
Beta-blocker	6,669 (56.9)	1,786 (56.7)	4,883 (57.0)	0.721
Angiotensin blockade	6,927 (59.1)	1,871 (59.4)	5,056 (59.1)	0.764
Calcium-channel blocker	2,817 (24.0)	720 (22.8)	2,097 (24.5)	0.064
Statin	10,379 (88.6)	2,743 (87.0)	7,636 (89.2)	0.001
Proton pump inhibitor	1,991 (17.0)	498 (15.8)	1,493 (17.4)	0.036

Values are n (%), mean ± SD, or median (Q1-Q3).

ACC = American College of Cardiology; AHA = American Heart Association; CABG = coronary artery bypass graft; DAPT = dual antiplatelet therapy; GFR = glomerular filtration rate; HbA_1c_ = hemoglobin A_1c_; HDL = high-density lipoprotein; LDL = low-density lipoprotein; LV = left ventricular; MDRD = Modification of Diet in Renal Disease; MI = myocardial infarction; PCI = percutaneous coronary intervention; PRU = P2Y_12_ reaction unit; WBC = white blood cell.

### Effect of procedural complexity

**Figure 2:**
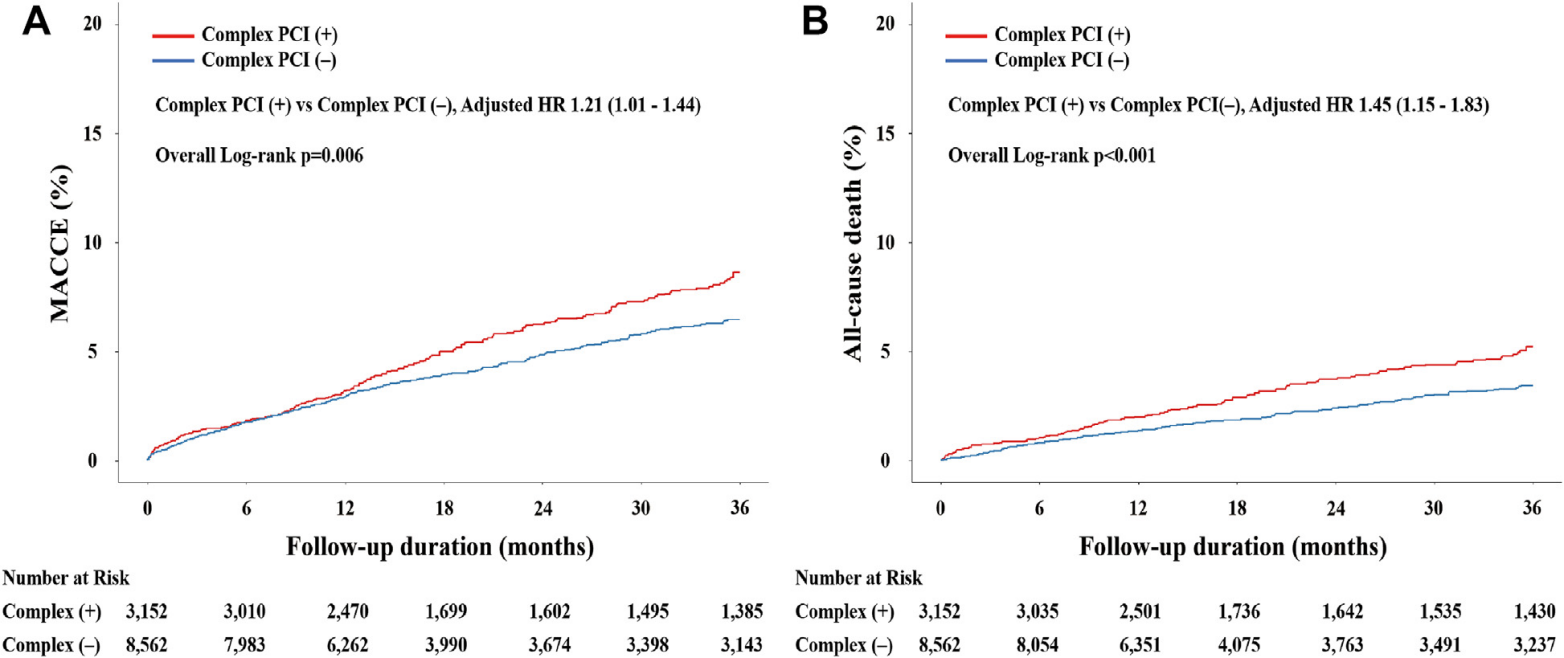
Kaplan-Meier Curves for MACCE and Death According to Complex PCI Kaplan-Meier estimates were conducted between the 2 groups for (A) MACCE and (B) death according to the complexity of PCI. Bonferroni’s correction was applied for multiple comparisons between the 2 groups. Abbreviations as in Figure 1.

At a median follow-up of 494 days (Q1-Q3: 364-1,773 days), patients who underwent C-PCI had higher crude rates of MACCE (HR_adjusted_: 1.21; 95% CI: 1.01-1.44) and all-cause death (HR_adjusted_: 1.45; 95% CI: 1.15-1.83) compared with those who underwent non–C-PCI (Figure 2), but the other adverse events did not reach statistical significance between the groups (Table 2). Using the landmark analysis (Supplemental Table 1, Supplemental Figure 1), we evaluated the association between PCI phenotype and clinical events over time. Within 1 year, complexity of PCI was only associated with the rate of all-cause death (HR_adjusted_: 1.40; 95% CI: 1.02-1.92). Between a post-PCI duration of 1 year and 3 years, the C-PCI phenotype showed a significant impact on the risk of MACCE (HR_adjusted_: 1.48; 95% CI: 1.14-1.92) and all-cause death (HR_adjusted_: 1.50; 95% CI: 1.06-2.11).

**Table 2:** Incidence of Clinical Outcomes According to Procedural Complexity

	Complex PCI (+) (n = 3,152)	Complex PCI (−) (n = 8,562)	Unadjusted HR (95% CI)	*P* Value	Adjusted HR (95% CI)	*P* Value
MACCE	192 (6.1)	378 (4.4)	1.27 (1.07-1.52)	0.007	1.21 (1.01-1.44)	0.035
All-cause death	116 (3.7)	189 (2.2)	1.53 (1.21-1.93)	0.000	1.45 (1.15-1.83)	0.002
Myocardial infarction	39 (1.2)	104 (1.2)	0.94 (0.65-1.35)	0.729	0.93 (0.64-1.35)	0.710
Stent thrombosis, definite	17 (0.5)	45 (0.5)	1.02 (0.59-1.79)	0.938	0.99 (0.57-1.74)	0.980
Stroke	49 (1.6)	96 (1.1)	1.29 (0.92-1.84)	0.143	1.17 (0.83-1.66)	0.371
Major bleeding	89 (2.8)	191 (2.2)	1.21 (0.94-1.56)	0.137	1.16 (0.9-1.49)	0.261

Values are n (%) unless otherwise indicated.

MACCE = major adverse cardiac and cerebrovascular event(s); PCI = percutaneous coronary intervention.

### Clinical outcomes according to platelet reactivity and procedural complexity

**Figure 3:**
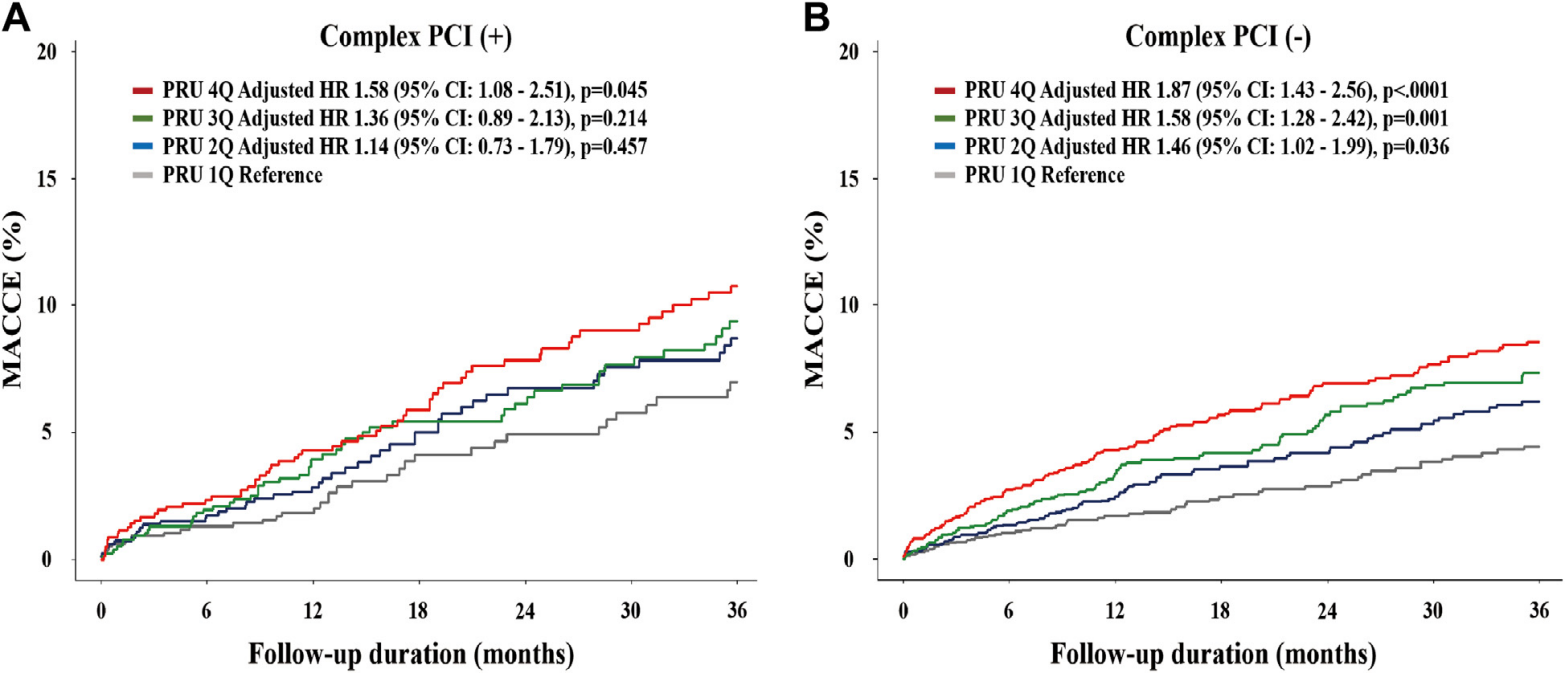
Incidence Rate of MACCE According to Quartile Distribution of PRU Incidence rate of MACCE between complex PCI (A) and noncomplex PCI (B) according to quartile distribution of P2Y_12_ reaction unit (PRU). Bonferroni’s correction was applied for multiple comparisons between the 2 groups. Abbreviations as in Figure 1.

The incidence rate of MACCE during the follow-up period according to the quartile distribution of PRU is presented in Figure 3. The fourth quartile group of PRU showed the highest risk of MACCE in the C-PCI group (HR_adjusted_ for the fourth vs first group: 1.58; 95% CI: 1.08-2.51; *P* = 0.045), whereas the incidence of MACCE increased according to the quartile category of PRU in the non–C-PCI group.

**Figure 4:**
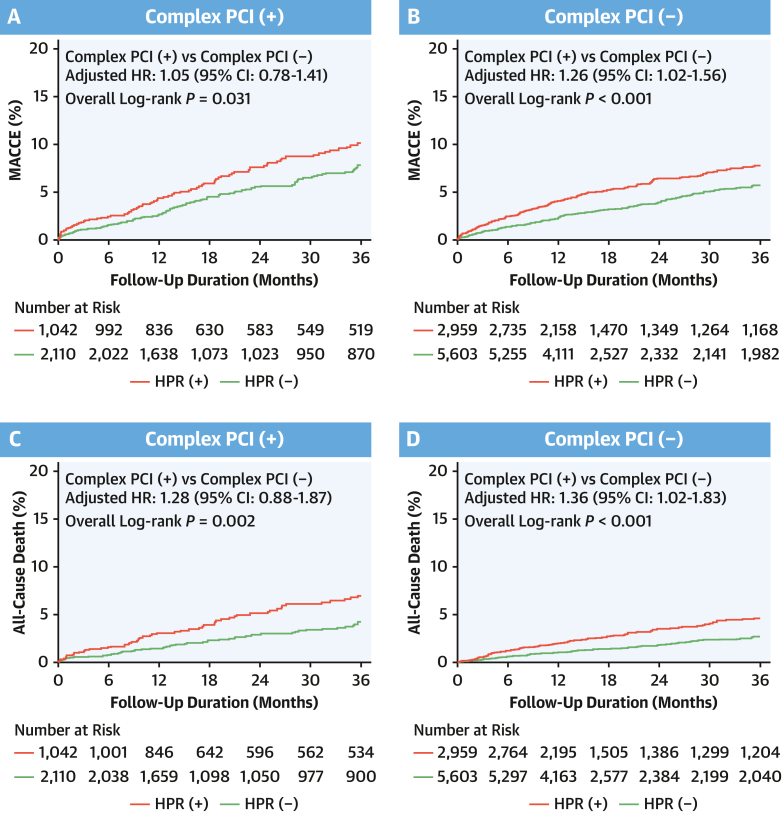
Kaplan-Meier Curves for MACCE and Death by PCI and HPR Kaplan-Meier estimates were conducted among the 4 groups for (A) MACCE and (B) death according to complex PCI and HPR. Bonferroni’s correction was applied for multiple comparisons among the 4 groups. Abbreviations as in Figure 1.

We categorized the enrolled patients into the groups based on the procedural complexity and HPR phenotype. The rates of MACCE and all-cause mortality increased proportionally according to C-PCI and HPR phenotype (Figure 4). Irrespective of procedural complexity, the impact of HPR criteria on clinical events appeared similar across each event (Table 3), in which ST appeared to be the most platelet-centric event (by an approximate 3-fold increase). In addition, the prognostic implications of HPR seemed to be more prominent within 1 year (Supplemental Table 2, Supplemental Figure 2). The rate of major bleeding did not differ between the groups (Supplemental Table 3, Supplemental Figure 3).

**Table 3:** Incidence of Clinical Outcomes According to Complex PCI and HPR

	Complex PCI (+)				Complex PCI (−)				*P* Value for Interaction
	HPR (+) (n = 1,042)	HPR (−) (n = 2,110)	Adjusted HR (95% CI)	*P* Value	HPR (+) (n = 2,959)	HPR (−) (n = 5,603)	Adjusted HR (95% CI)	*P* Value	
MACCE	81 (7.8)	111 (5.3)	1.05 (0.78-1.41)	0.755	169 (5.7)	209 (3.7)	1.26 (1.02-1.56)	0.032	0.731
All-cause death	56 (5.4)	60 (2.8)	1.28 (0.88-1.87)	0.203	94 (3.2)	95 (1.7)	1.36 (1.02-1.83)	0.039	0.978
Myocardial infarction	15 (1.5)	24 (1.1)	0.95 (0.482-1.856)	0.872	41 (1.4)	63 (1.1)	1.12 (0.74-1.68)	0.594	0.938
Stent thrombosis	10 (1.0)	7 (0.3)	3.16 (1.16-8.58)	0.024	27 (0.9)	18 (0.3)	3.06 (1.65-5.68)	<0.001	0.411
Stroke	17 (1.6)	32 (1.5)	0.84 (0.46-1.53)	0.565	36 (1.2)	60 (1.1)	1.02 (0.66-1.55)	0.945	0.849
Major bleeding	34 (3.3)	55 (2.6)	1.02 (0.66-1.59)	0.922	73 (2.5)	118 (2.1)	0.91 (0.67-1.23)	0.525	0.814

Values are n (%) unless otherwise indicated.

HPR = high platelet reactivity; other abbreviations as in Table 2.

**Figure 5:**
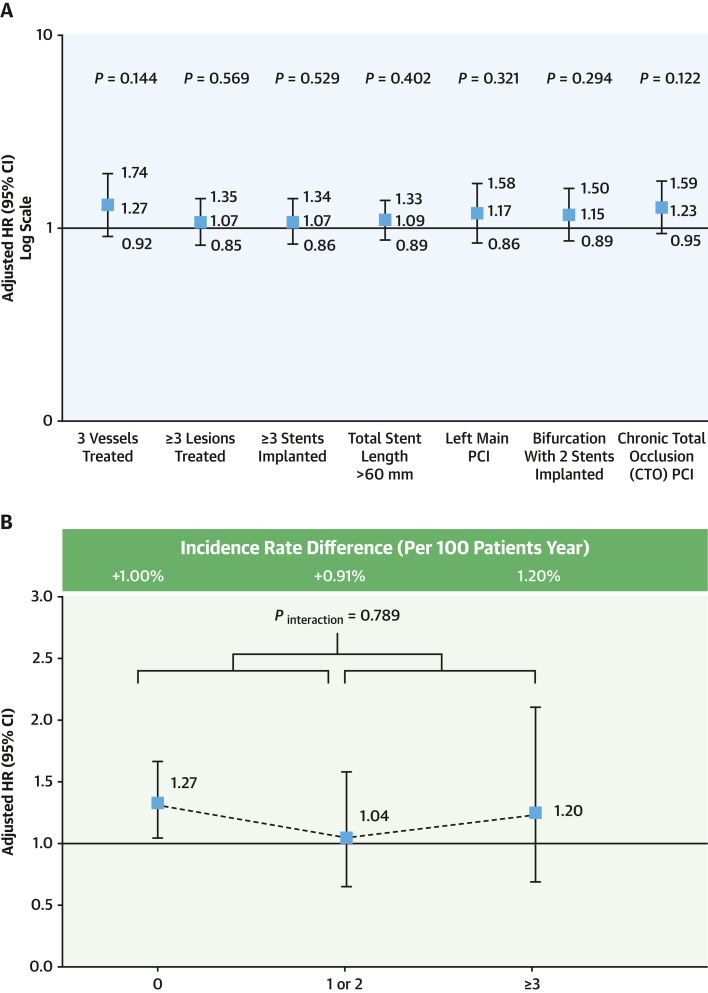

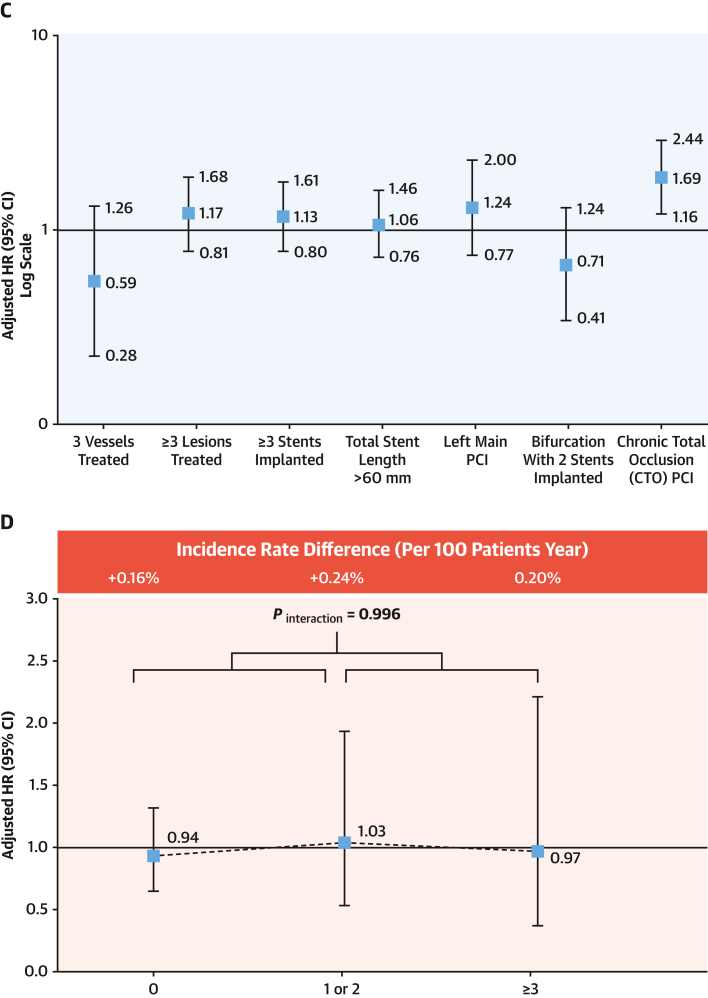
Impacts of HPR on MACCE and Major Bleeding (A and C) subtype of complex PCI and (B and D) the extent of procedural complexity. Abbreviations as in Figures 1 and 2.

The adjusted impact of HPR on the occurrence of MACCE and major bleeding according to the type of C-PCI is illustrated in Figures 5A and 5C. Each subset of complex PCI modestly increased the risk of MACCE. Using a multivariate Cox proportional hazard regression model, we evaluated the clinical impact of HPR phenotype on the occurrence of MACCE and major bleeding according to the number of procedural complexities (Figures 5B and 5D). There were no significant interactions between the prognostic implications in terms of the extent of procedural complexity.

## Discussion

This subanalysis using a PTRG-PFT set investigated the long-term prognostic implication of HPR according to procedural complexity in DES-treated East Asian patients. The principal findings are as follows: 1) C-PCI phenotype increased the risk of MACCE and all-cause death during the 3-year clinical follow-up; 2) HPR criteria were significantly associated with MACCE occurrence, irrespective of procedural complexity; 3) the prognostic implication of HPR was prominently related to ST; and 4) the relative contribution of HPR toward clinical events did not significantly change according to the type or extent of procedural complexity.

### The prognostic implications of complex PCI

Procedural complexity has been hypothesized to increase ischemic risk, especially when multiple complexity features are present.^3^ However, the prognostic implications can vary according to the time interval after PCI and the characteristics of the patients studied.

Giustino et al^3^ reported that patients who underwent C-PCI had a higher incidence of coronary thrombotic events, but this feature was not associated with an increased risk of major bleeding during 1-year follow-up. The e-Ultimaster registry (Prospective, Single-arm, Multi Centre Observations Ultimaster DES Registry) also showed that C-PCI phenotype increased the risk of 1-year cardiac death and complications compared with simple PCI.^20^ A subanalysis from the ISAR-REACT (Intracoronary Stenting and Antithrombotic Regimen: Rapid Early Action for Coronary Treatment) trial (3,377 ACS patients) reported that procedural complexity was significantly associated with an increased incidence of ischemic events (HR_adjusted_: 1.44; 95% CI: 1.14-1.82; *P* = 0.002) at 1 year after the procedure.^21^ Clinical evidence suggests that the prognostic implication of procedural complexity is similar regardless of the disease entity (ACS vs stable angina).

Several clinical studies in East Asian countries have shown conflicting results on this issue. Endo et al^22^ reported that patients who underwent complex DES implantation did not show differences in the risk of all-cause death (log-rank *P* = 0.12) and MACCE (log-rank *P* = 0.64) during 3-year follow-up (1,062 DES-treated patients). In addition, another Korean study also showed that the clinical outcomes did not differ according to lesion complexity for up to 2 years after the current DES implantation (N = 926)^23^ However, these studies enrolled relatively small numbers of participants. A largescale Korean registry study (N = 13,172) has supported the close relationship between procedural complexity and clinical events, showing that clinical and procedural factors were both significant predictors of MACCE within the second year.^24^ In addition, the present PTRG-DES consortium (N = 11,714) showed that procedural complexity was associated with long-term clinical events.

### Impact of HPR according to procedural complexity

In the setting of complex PCI cases, the use of a potent antiplatelet regimen and/or prolonged use of DAPT with moderate P2Y_12_ inhibition would be worth considering to prevent the occurrence of thrombotic events, which may be related to the close interaction between anatomical/procedural complexity and thrombogenic components.^25^ However, recent evidence has suggested conflicting findings for an optimal strategy to overcome this accompanying risk following C-PCI.

The prognostic implication of HPR or type of P2Y_12_ inhibitor according to procedural complexity has not been extensively investigated in patients treated with the current-generation DES. In a subanalysis of the ADAPT-DES (Assessment of Dual Antiplatelet Therapy With DES) trial (N = 8,582), there was no statistical interaction between HPR and bifurcation PCI regarding the risk of target vessel failure (adjusted *P*_*interaction*_ = 0.87). In addition, bifurcation PCI was associated with a higher risk of 2-year adverse ischemic events than nonbifurcation PCI, a risk that is particularly high when both bifurcation branches are stented, and with HPR conferring similar risk for bifurcation and nonbifurcation PCI.^26^

On the contrary, there are some reports that procedural complexity itself would increase the clinical benefit with the use of a potent P2Y_12_ inhibitor. A subanalysis from the multicenter observational study comparing clopidogrel vs prasugrel in acute coronary syndrome patients undergoing PCI (PROMETHEUS) compared clinical outcomes during clopidogrel vs prasugrel treatment in ACS patients undergoing PCI (N = 19,914).^11^ Compared with clopidogrel, prasugrel significantly decreased the risk of 1-year MACE for C-PCI (HR_adjusted_: 0.79; 95% CI: 0.68-0.92), but not for non–C-PCI (HR_adjusted_: 0.91; 95% CI: 0.77-1.08), albeit there was no evidence of interaction (*P*_interaction_ = 0.281).

Prolonged DAPT administration may be applicable to overcome long-term atherothrombotic events following procedural complexity. In the patient-level analysis of 6 randomized controlled trials investigating post-PCI DAPT duration (N = 9,577),^3^ long-term DAPT (≥12 months) yielded significant reductions in MACCE in C-PCI (HR_adjusted_: 0.56; 95% CI: 0.35-0.89) vs non–C-PCI (HR_adjusted_: 1.01; 95% CI: 0.75-1.35) (*P*_interaction_ = 0.01) compared with short-term DAPT (3-6 months). However, the post hoc analysis of the DAPT study suggested a contrary result showing similar benefit against MI and ST during 30- vs 12-month DAPT for patients with (HR_adjusted_: 0.55; 95% CI: 0.38-0.79; *P* = 0.001) and without (HR_adjusted_: 0.52; 95% CI: 0.39-0.69; *P* < 0.001) anatomical complexity (*P*_interaction_ = 0.81).^27^ Another recent pooled patient-level study showed that P2Y_12_ inhibitor monotherapy after 1- to 3-month DAPT vs standard DAPT (approximately 70% of the cohort: treated with potent P2Y_12_ inhibitor) was associated with similar rate of ischemic events and lower risk of major bleeding, irrespective of PCI complexity.^28^

Taken together, the clinical evidence supports the notion that potent P2Y_12_ inhibition is required to overcome ischemic risk related to the thrombogenic milieu, and early aspirin discontinuation combined with potent P2Y_12_ inhibition could be applicable even for these cases. Therefore, an optimized and individualized DAPT strategy could be required to prevent atherothrombotic events in patients with C-PCI features.

### Study Limitations

The PTRG-DES consortium included only DES-treated patients receiving clopidogrel treatment. Therefore, the analysis could not suggest a direct comparison regarding clopidogrel vs potent P2Y_12_ inhibitors according to procedural complexity. Second, it is known that platelet reactivity can vary over time according to the disease type and phase. However, PFT was performed once during PCI in the present study. Third, this analysis could not cover the effect of early aspirin discontinuation in relation to PCI complexity^28^ because the PTRG-DES consortium showed a high prevalence of prolonged DAPT maintenance in Korean society (DAPT maintenance: 535 ± 355 days in the present analysis). The issue regarding the clinical impact of early aspirin discontinuation according to potency of P2Y_12_ inhibition is an important topic to be explored. Finally, the number of patients might be insufficient to observe the effect of HPR on clinical events in each component in complex PCI. Therefore, the possibility of insufficient power in the statistical analysis should be considered.

## Conclusions

In this largescale East Asian cohort, complex PCI was significantly associated with 3-year rates of MACCE and all-cause death. The HPR phenotype increased the risk of atherothrombotic events, but its prognostic implication appears similar irrespective of the type and extent of procedural complexity. This finding may support the clinical benefit of potent P2Y_12_ inhibition being primarily related to the presence of the HPR phenotype, and having a lower association with procedural complexity.

Perspectives **COMPETENCY IN MEDICAL KNOWLEDGE:** Complex PCI increased 3-year rates of MACCE and all-cause death compared with non-complex PCI after stenting. Although HPR phenotype increased the risk of atherothrombotic events, the prognostic implications appear similar irrespective of the type and extent of procedural complexity.

**COMPETENCY IN PATIENT CARE:** Potent P2Y_12_ inhibition may be necessary to overcome ischemic risk related to factors in the thrombogenic milieu such as HPR phenotype in complex PCI patients.

**TRANSLATIONAL OUTLOOK:** Large randomized trials are necessary to prove the benefit of dose escalation for antiplatelet therapy to reduce ischemic risk in complex coronary artery disease patients undergoing PCI.

## Funding Support and Author Disclosures

The study was designed by the principal investigator and executive committee, and was sponsored by the Korean Society of Interventional Cardiology. Dr Jeong has received honoraria for lectures for AstraZeneca, Daiichi-Sankyo, Sanofi, Hanmi Pharmaceuticals, and Yuhan Corporation; and research grants or support from Yuhan Corporation and U&I Corporation. Dr Joo has received honoraria for lectures for AstraZeneca, Hanmi, Samjin, Dong-A, HK inno, N Pharmaceuticals, and DIO Medical Ltd. Dr Song has received honoraria for lectures for AstraZeneca, Daiichi-Sankyo, Sanofi, Bayer Korea, and Samjin Pharmaceutical. All other authors have reported that they have no relationships relevant to the content of this paper to disclose.
