# Supplementary material for: Prognostic Implication of Platelet Reactivity According to Procedural Complexity After PCI: Subanalysis of PTRG-DES Consortium
Source: JACC Asia. 2023 Dec 26;4(3):185–98. doi: 10.1016/j.jacasi.2023.10.011 (PMC10920055; doi:10.1016/j.jacasi.2023.10.011)
Supplement: Supplemental Tables 1–3 and Supplemental Figures 1–3 [file mmc1.docx]

**Prognostic Implication of Platelet Reactivity According to Procedural Complexity After PCI: Sub-analysis of PTRG-DES Consortium**

Xuan Jin, MD, Young-Hoon Jeong, MD, Kwang Min Lee, PhD, Sung Cheol Yun, PhD, Byeong-Keuk Kim, MD, Hyung Joon Joo, MD, Kiyuk Chang, MD, Yong Whi Park, MD, Young Bin Song, MD, Sung Gyun Ahn, MD, Jung-Won Suh, MD, Sang Yeub Lee, MD, Jung Rae Cho, MD, Ae-Young Her, MD, Hyo-Soo Kim, MD, Do-Sun Lim, MD, Eun-Seok Shin, MD, Moo Hyun Kim, MD, On Behalf of the PTRG-DES Consortium Investigators

**Supplemental Table 1. Prognostic implications of complex PCI for MACCE and all-cause death: Landmark Analysis**

|  | | Adjusted HR (95% CI) | p |
| --- | --- | --- | --- |
| MACCE | 1 year | 1.03 (0.81 - 1.30) | 0.832 |
|  | 1–3 year | 1.48 (1.14 - 1.92) | 0.004 |
| Death | 1 year | 1.40 (1.02 - 1.92) | 0.039 |
|  | 1–3 year | 1.50 (1.06 - 2.11) | 0.022 |

CI = confidence interval; HR = hazard ratio; MACCE = major adverse cardiac and cerebrovascular events; PCI = percutaneous coronary intervention.

**Supplemental Table 2. Prognostic implications of HPR for MACCE and all-cause death according to complex PCI: Landmark Analysis.**

|  | | **Complex PCI(+)** | | **Complex PCI(-)** | |
| --- | --- | --- | --- | --- | --- |
|  |  | Adjusted HR (95% CI) | p | Adjusted HR (95% CI) | p |
| MACCE | 1 year | 1.38 (1.06 - 1.83) | 0.012 | 1.46 (1.12 - 1.91) | 0.005 |
|  | 1–3 year | 0.95 (0.62 - 1.45) | 0.810 | 0.98 (0.69 - 1.38) | 0.890 |
| Death | 1 year | 1.73 (1.08 - 3.01) | 0.009 | 1.41 (1.05 - 2.09) | 0.018 |
|  | 1-3 year | 1.14 (0.66 - 1.97) | 0.631 | 1.57 (1.02 - 2.45) | 0.029 |

CI = confidence interval; HPR = high platelet reactivity; HR = hazard ratio; MACCE = major adverse cardiac and cerebrovascular events; PCI = percutaneous coronary intervention.

**Supplemental Table 3. Incidence of adjusted models of major bleeding according to complex PCI and HPR status.**

| Group | Adjusted HR (95% CI) | p |
| --- | --- | --- |
| C-PCI(+) & HPR(+) | 1.10 (0.75 - 1.62) | 0.634 |
| C-PCI(+) & HPR(-) | 1.13 (0.82 - 1.56) | 0.463 |
| C-PCI(-) & HPR(+) | 0.92 (0.68 - 1.23) | 0.562 |
| C-PCI(-) & HPR(-) | Reference |  |

CI = confidence interval; HPR = high platelet reactivity; HR = hazard ratio; PCI = percutaneous coronary intervention.

**Supplemental Figure 1.** **Landmark analysis for (A) MACCE and (B) all-cause death according to PCI complexity**


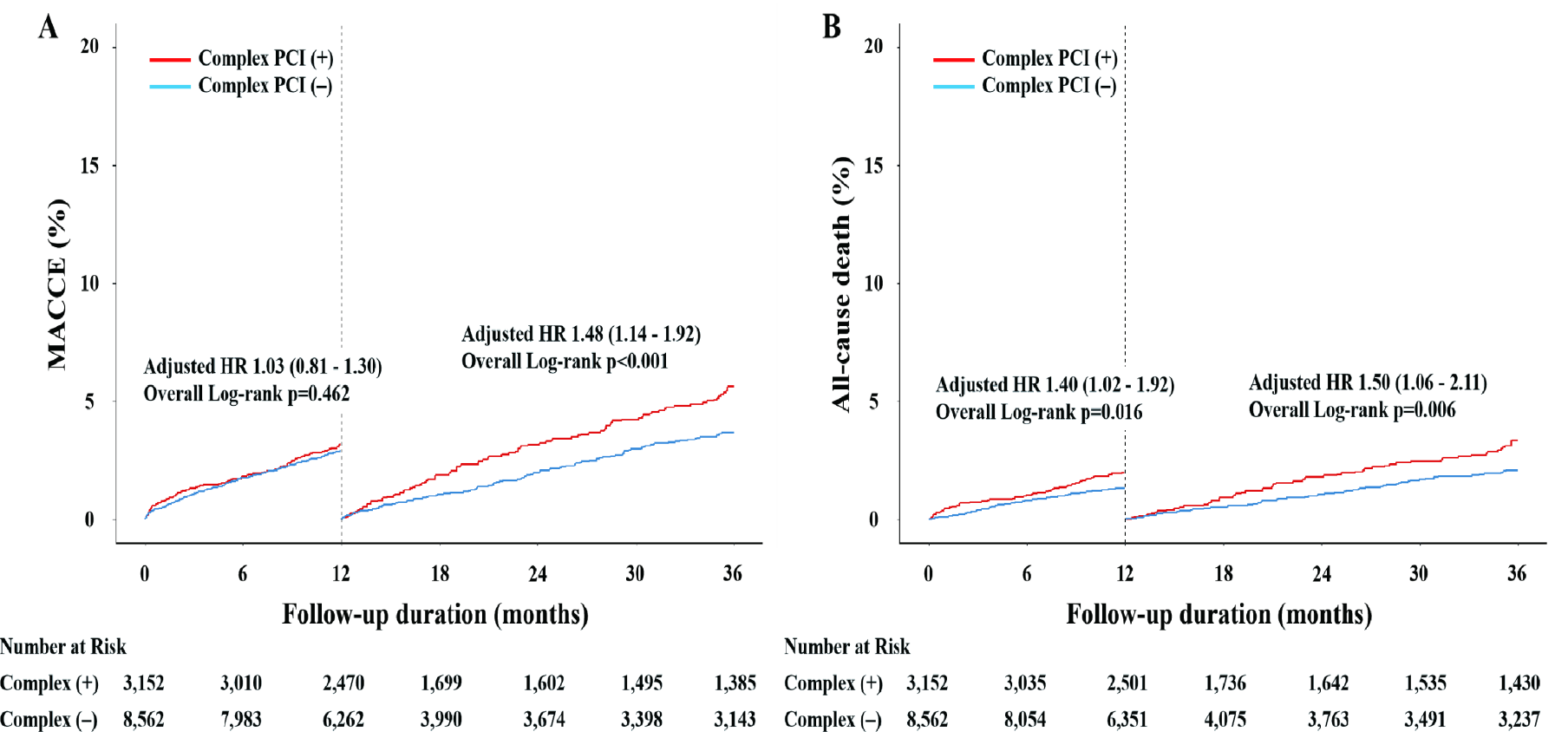


CI = confidence interval; HR = hazard ratio; MACCE = major adverse cardiac and cerebrovascular events; PCI = percutaneous coronary intervention.

Kaplan-Meier estimates were conducted between two groups according to complexity of PCI. Bonferroni’s correction was applied for multiple comparisons between the two groups.**Supplemental Figure 2. 1-year landmark analysis for (A) MACCE and (B) all-cause death according to PCI complexity and HPR status**


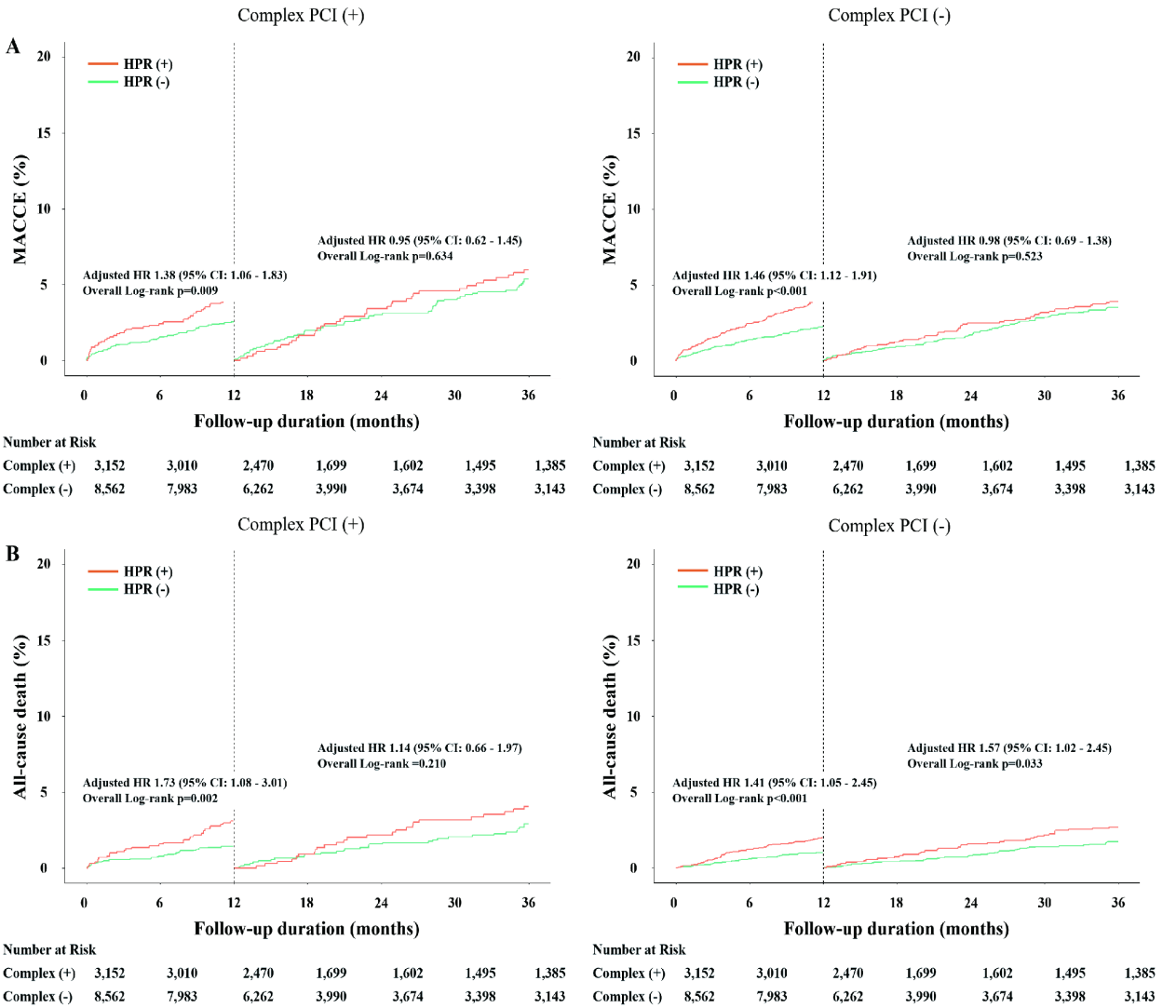


CI = confidence interval; HPR = high platelet reactivity; HR = hazard ratio; MACCE = major adverse cardiac and cerebrovascular events; PCI = percutaneous coronary intervention.

Kaplan-Meier estimates were conducted between two groups according to HPR in both the non-C-PCI arm and C-PCI arm. Bonferroni’s correction was applied for multiple comparisons between the two groups.

**Supplemental Figure 3. Incidence of major bleeding according to PCI complexity and HPR status**


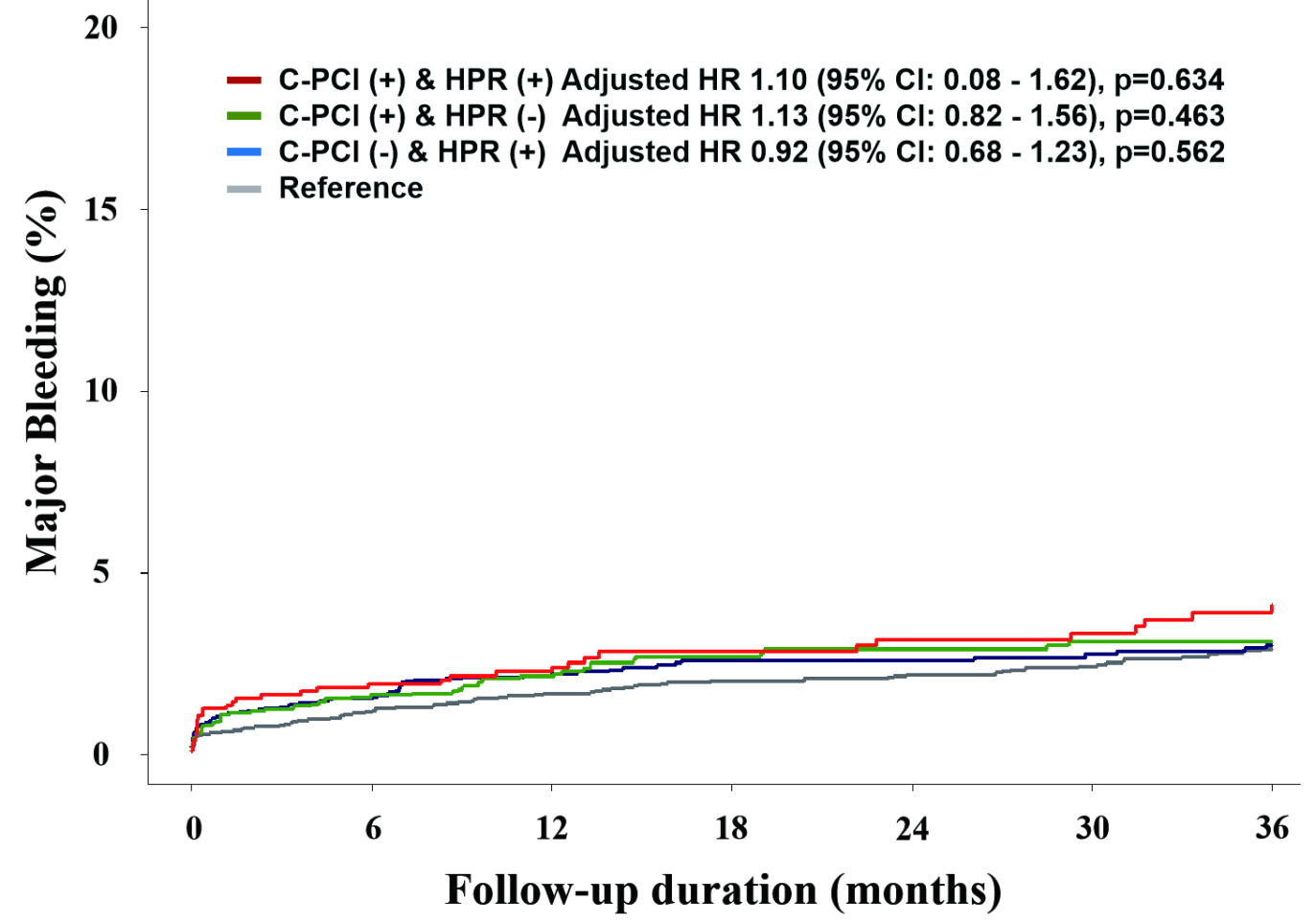


CI = confidence interval; HPR = high platelet reactivity; HR = hazard ratio; PCI = percutaneous coronary intervention.

Kaplan-Meier estimates were conducted between two groups according to HPR in both the C-PCI arm and non-C-PCI arm. Bonferroni’s correction was applied for multiple comparisons between the two groups.
